# Costing analysis of field implementation of hepatitis C case detection in rural Maung Russey operational district, Cambodia

**DOI:** 10.5365/wpsar.2020.11.3.006

**Published:** 2021-07-12

**Authors:** Su Myat Han, Ir Por, Keo Samley, Voeurng Bunreth, Chris Smith, Koya Ariyoshi, Jean-Philippe Dousset, Mickael Le Paih

**Affiliations:** a Médecins Sans Frontières – France, Phnom Penh, Cambodia.; b Department of Tropical Medicine and Global Health, Nagasaki University, Japan.; c National Institute of Public Health, Cambodia.; d Communicable Disease Control Department, Ministry of Health, Cambodia.; e Provincial Health Department, Battambang, Ministry of Health, Cambodia.; f Department of Clinical Medicine, Nagasaki University, Japan.

## Abstract

**Background:**

When a new health programme is introduced, it is crucial to estimate the costs for rational health policy decision-making. The aim of this study was to determine the costs of implementing two strategies for hepatitis C virus (HCV) screening in rural Cambodia.

**Methods:**

We retrospectively analysed clinical and cost data that were collected routinely for a demonstration project for scaling up HCV screening and testing in Cambodia. The programme data were collected between March and December 2018 in Maung Russey operational district in Battambang Province, Cambodia.

**Findings:**

During the study period, 24 230 people were screened; 1194 (5%) were HCV seropositive, of whom 793 (66%) were confirmed to be viraemic. During the study period, 18% of the estimated population of the operational district were screened, of whom 45% were estimated to be seropositive and 41% to be viraemic. With passive screening alone, 8% of the estimated population were screened, of whom 29% were estimated to be seropositive and 28% viraemic. The cost per detected viraemic case was US$ 194 for passive screening alone and US$ 283 for passive and active screening combined. Labour costs (31%) and tests and materials (29%) comprised the largest proportions of the cost.

**Conclusion:**

Combined active and passive screening per viraemic case detected was US$ 89 more expensive than passive screening alone but provided a higher yield (41% versus 28%) of viraemic cases. Therefore, adding active screening to passive screening is beneficial. Selective active screening strategies, such as targeting people over 45 years and other higher-risk groups, added value for HCV diagnosis.

An estimated 71 million people globally are living with chronic hepatitis C virus (HCV) infection, but < 20% of these individuals are aware of their infection status. (*1*) Although affordable directly acting antivirals are available to treat HCV, (*2*) finding cases remains difficult, obviating increasing access to HCV treatment, especially in low- and middle-income countries such as Cambodia.

It has been estimated that about 2% of the population of Cambodia is living with chronic HCV infection, with a higher prevalence in older age groups. (*3*-*6*) Genotypes 1 and 6 were the main HCV genotypes in the country, each at about 46% prevalence. (*7*) Liver cancer is the leading cause of cancer deaths in the country, with 76% attributed to hepatitis B and/or HCV infection. (*8*, *9*) In a seroprevalence survey conducted in Maung Russey health district in Cambodia, about 65% of the population was unaware of HCV. (*6*)

In early 2020, the Cambodia Ministry of Health established a national strategic plan (2020–2024) and national guidelines for viral hepatitis B and C, (*10*) which includes scaling up HCV testing nationwide; however, plans for screening have not yet been issued. Recommendations on the cost and effectiveness of different screening strategies for HCV are therefore necessary to ensure effective HCV service delivery and management in Cambodia and in countries with similar settings. Population or mass screening has been used in countries such as Egypt and Rwanda; (*11*, *12*) however, high costs are a potential drawback to universal programmes.

In Cambodia, the medical humanitarian organization Médecins Sans Frontières (MSF) has been providing free HCV screening and treatment in collaboration with the Ministry of Health in Phnom Penh since September 2016 and in Battambang Province since March 2018. The projects were managed in partnership with the Department of Communicable Disease Control. By the end of 2020, 130 327 people had been screened in Cambodia, of whom 30 684 (22%) were found to be HCV seropositive; of those, 20 645 (70%) were HCV viraemic, and 18 900 had initiated treatment with directly acting antivirals.

The aim of the current study was to evaluate the cost and effectiveness of two screening strategies to inform public health authorities for future national screening.

## Methods

### Study setting

Data routinely collected for an HCV programme in Maung Russey, Cambodia, between March and December 2018 were analysed. Maung Russey is a rural operational district with an estimated population of 209 949 distributed in 175 villages in 2018. An MSF project for HCV screening and treatment was launched with existing Ministry of Health services, which included 13 health centres and a referral hospital covering the entire Maung Russey district.

### Screening strategies

Two screening strategies were used to identify HCV cases in the community: passive and active screening. Passive screening covered the general population aged (*3*)18 years and comprised voluntary HCV testing at health centres. This was initiated in March 2018 and continued for the duration of the study period.

In September 2018, after six months of passive screening and to increase case detection, active screening was added. Active screening targeted people aged (*3*)45 years, as epidemiological studies indicated that this age group is at higher risk of HCV infection than others. (*4*, *6*)

The implementation team comprised an MSF nurse, health centre staff, village chiefs and community volunteers. Information on screening activities and awareness about HCV were provided to the community before HCV screening days by village and community leaders through a village information-sharing system for one half day. On the screening day, all seropositive cases were referred to their closest health centre for confirmatory testing of viral load. The active screening strategy comprised one day of screening per village between September and December 2018 in the 175 villages and subvillages in the Maung Russey operational district. Passive screening continued during this time.

In both strategies, screening of people who were under tuberculosis (TB) treatment was postponed until the end of their treatment. People known to be living with HIV were referred to the National Centre for HIV/AIDS, Dermatology and Sexually Transmitted Diseases. Screening of pregnant or breastfeeding women was deferred until the end of breastfeeding. There was no overlap of the passive and active programmes, as all people were systematically asked by the screening team whether they had already been screened and/or treated for HCV infection.

### Screening test and diagnosis

The rapid diagnostic test SD Bioline® was used for diagnosis in both screening strategies on capillary blood collected by fingerprick by trained nurses. Positive cases were considered HCV seropositive. Confirmatory testing was performed with a viral load test based on a polymerase chain reaction assay for HCV RNA. Cases positive in this test were considered viraemic positive cases.

From seropositive patients detected in the passive screening programme, venous blood was collected for a viral load test at a health centre. Seropositive patients detected by active screening were referred to the nearest health centre for blood collection. The 2% of referred patients who did not show up for the viral load test were actively followed up by a phone call from an MSF nurse.

The collected blood samples were transported to the referral hospital for confirmatory assays. Collected specimens were centrifuged on the same day and transported via cold chain (2–8 °C) to the Maung Russey hospital laboratory. Samples not processed for analysis within 24 hours were stored in a refrigerator (2–8 °C). The GeneXpert® HCV viral load assay was used.

### Screening effectiveness

The “number needed to screen” (NNS) for diagnosis of one viraemic case was calculated as the number of screened people divided by the number of confirmed HCV viraemic cases.

To compare the effectiveness of the two screening strategies (passive versus passive plus active screening), three outcomes were assessed: (i) the number of serological tests performed as a percentage of the target population; (ii) the number of positive serological cases as a percentage of the estimated number of HCV cases derived from a previous seroprevalence survey; (*4*) and (iii) the number of viraemic cases as a percentage of the estimated number of HCV cases. The denominators of each outcome were as follows.

The targeted population was estimated from the 2013 Cambodia intercensal population survey. (*13*) The estimated targeted populations were 133 162 people aged (*3*)18 years and 41 823 aged (*3*)45 years.
The numbers of seropositive cases were estimated to be 2663 ((*3*)18 years) and 2133 ((*3*)45 years) in accordance with the results of a previous seroprevalence survey, for seropositivity rates of 2.6% ((*3*)18 years) and 5.1% ((*3*)45 years).
The numbers of viraemic cases were estimated to be 1943 ((*3*)18 years) and 1514 ((*3*)45 years) in accordance with the previous seroprevalence survey of a prevalence of viraemia of 1.9% in the general population and 3.6% among people (*3*)45 years.

### Costing analysis

Data were collected on expenditures for both passive and active screening, and data on the salaries of Ministry of Health and MSF staff involved in HCV screening were collected as a component of labour costs. Expenditure for screening included training and capacity-building, equipment (including depreciation), labour (for project personnel and health facility staff as opportunity costs), travel, materials and tests, user fees (MSF paid for uninsured patients to access other tests at the same time as HCV testing) and indirect costs considered to be associated with HCV screening, including overhead costs of the MSF office in Maung Russey, the Maung Russey referral hospital and health centres. The mean cost per HCV case identified was calculated by summing the cost of all resources used for screening and dividing them by the number of viraemic cases diagnosed. Costs are expressed in US dollars.

## Results

### Screening uptake

During the study period (March to December 2018), 24 230 adults ((*3*)18 years) were screened for HCV, of whom 1194 (5%) were seropositive. Among the seropositive cases, 793 (66%) were viraemic. While passive screening of the adult population ((*3*)18 years) was conducted of 9049 people between March and August 2018, combined active ((*3*)45 years) and passive ((*3*)18 years) screening covered 15 181 individuals between October and December 2018. A higher proportion of seropositive cases in the (*3*)45-year age group (12.4%) was detected with passive screening than with combined active and passive screening (3.7%); however, 14 448 people were screened with combined passive and active screening and only 4396 with passive screening. During the entire study period and with each of the screening modalities, the NNS for detecting one HCV viraemic case in the age group (*3*)45 years (11 for passive and 43 for active) was lower than that for the age group 18–44 years old (95 for passive and 56 for active) (**Table 1**).

**Table 1:** Outcomes of passive and active screening in Maung Russey operational district, Cambodia,  March–December 2018

Age group	Total screened	Seropositive cases		Viraemic cases		
	N	n	(% of total screened)	n	(% of seropositive cases)	NNS
Phase 1: Passive screening (March–August)						
18–44 years	4653	78	1.7%	49	62.8%	95
^3^45 years	4396	547	12.4%	391	71.5%	11
Total	9049	625	6.9%	440	70.4%	21
Phase 2: Passive and active screening (September–December)						
18–44 years	733	32	4.4%	13	40.6%	56
^3^45 years	14 448	537	3.7%	340	63.3%	43
Total	15 181	569	3.7%	353	62.0%	43
Both phases (March–December)						
Total	24 230	1194	4.9%	793	66.4%	31

NNS: number needed to screen.

### Screening effectiveness

**Figure 1:**
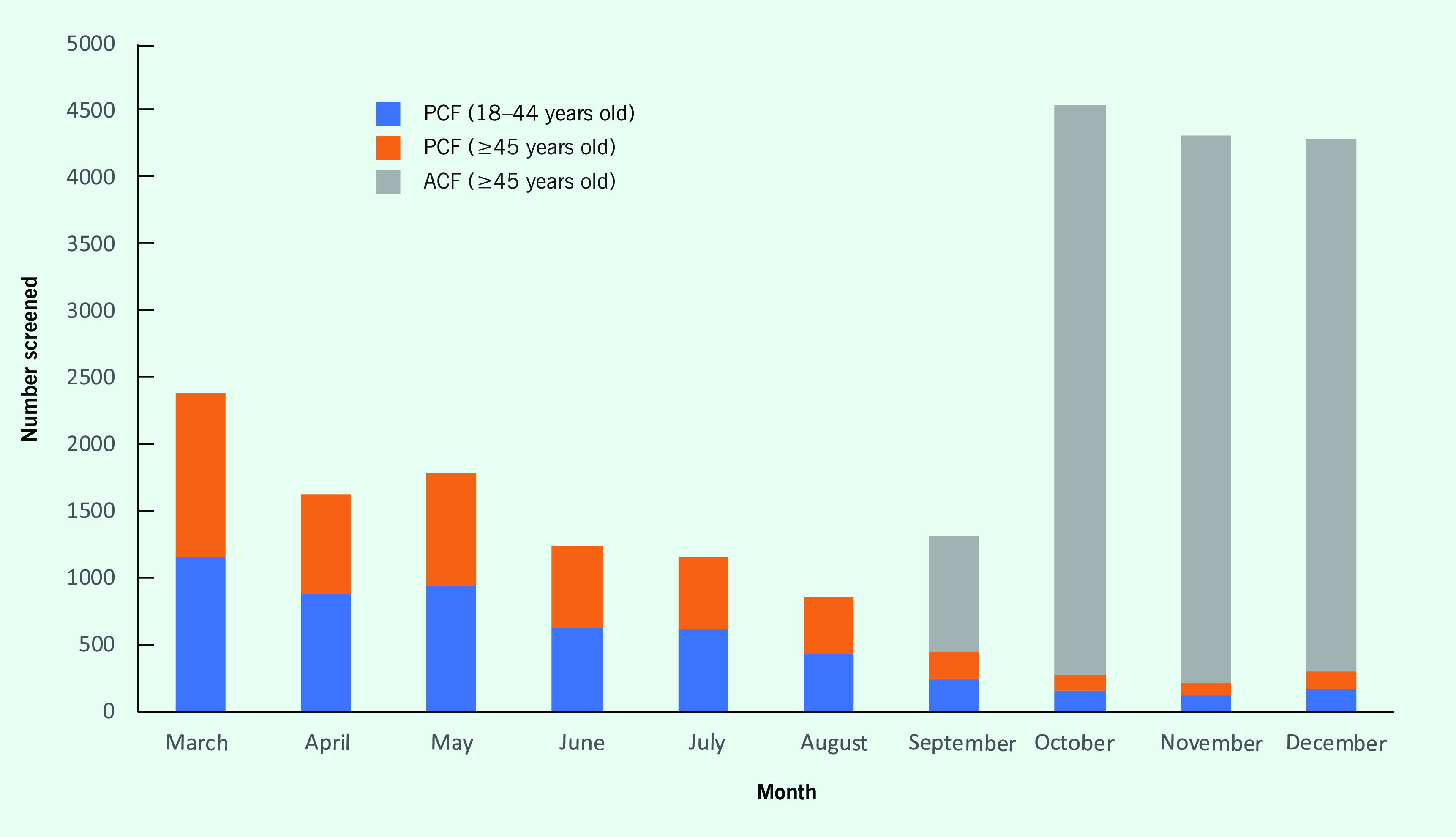
Numbers of cases screened in the passive and active screening programmes by age group and month, Maung Russey operational district, Cambodia, March–December 2018

**Figure 2:**
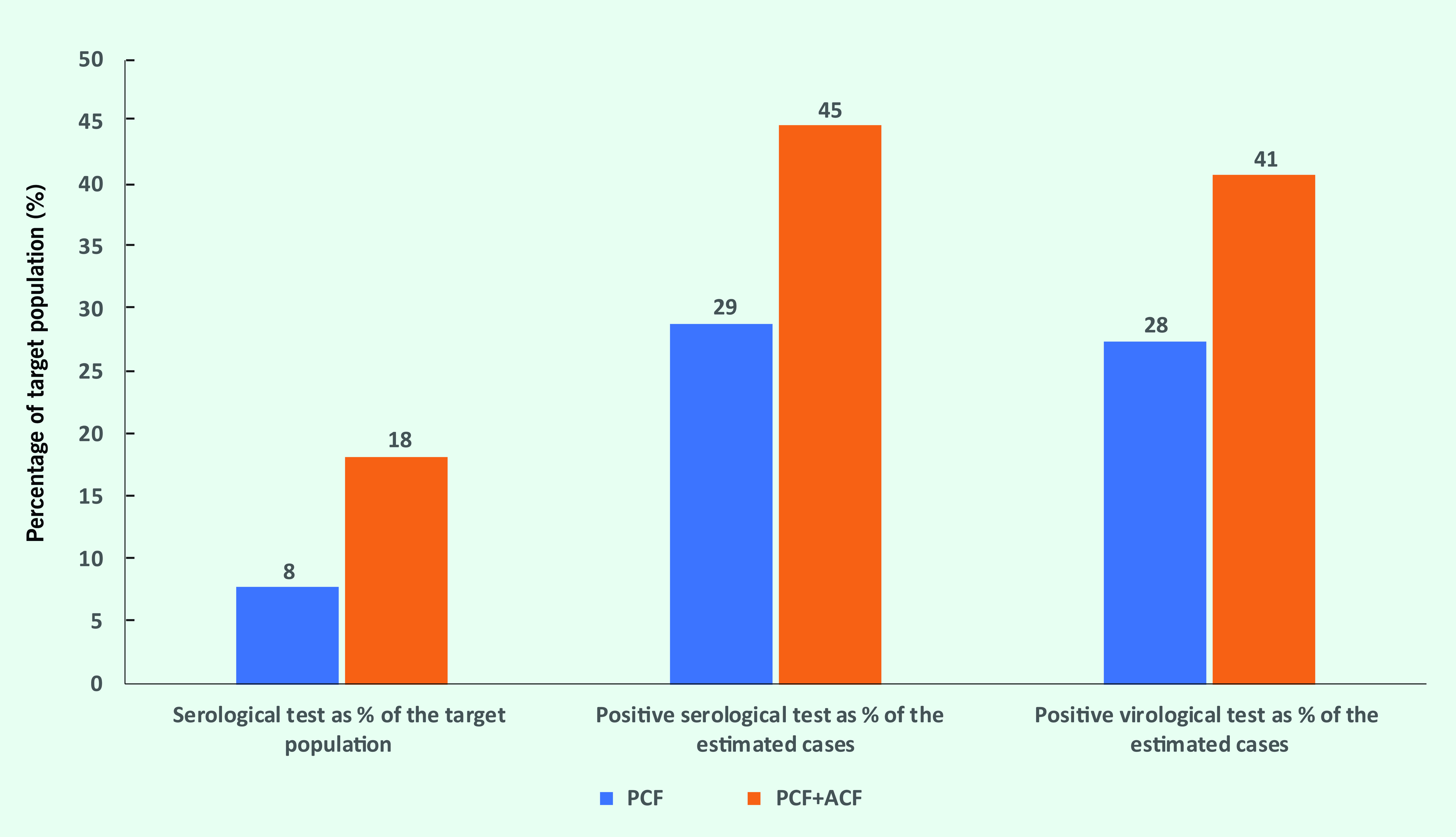
Effectiveness of passive and active screening in Maung Russey operational district, Cambodia, March–December 2018

**Fig. 1** shows the numbers of cases screened by age group and month in the passive and active screening programmes between March and December 2018. Combined passive and active screening covered 18% of the target population, while passive screening covered only 8% (**Fig. 2**). The proportions of seropositive and viraemic cases to the estimated number of cases were also higher for combined passive and active screening (45% and 41%, respectively) than for passive screening alone (29% and 28%, respectively).

[**insert** Figure 1]

[**insert** Figure 2]

### Cost of the screening programme

Between March and December, the programme cost US$ 85 208 for passive screening and US$ 99 733 for active and passive screening. The cost per viraemic positive case was US$ 194 for passive screening and US$ 283 for combined passive and active screening. The largest cost contributors were labour (31%), tests and materials (29%) and indirect costs (25%) (**Table 2**).

**Table 2:** Implementation costs of passive and active screening by cost category (US$, 2018) in Maung Russey operational district, Cambodia, March–December 2018

Cost items	Programme cost (US$)		Cost per case screened (US$)		Cost profile (%)
	Passive (Mar–Aug)	Passive + active (Sep–Dec)	Passive (Mar–Aug)	Passive + active (Sep–Dec)	
Training (*1*)	1632	1632	3.7	4.6	2
Field equipment (*2*)	3352	3179	7.6	9.0	3
Labour (*3*)	31 791	31 155	70.1	88.3	31
Transport (*4*)	18	11 790	0.0	33.4	9
Tests and materials (*5*)	24 906	28 526	56.6	80.8	29
User fees (*6*)	762	1439	1.7	2.0	1
Indirect costs (*7*)	22 747	22 747	51.7	64.4	25
Total cost	85 208	99 733	193.7	282.5	100

^1^ Cost of training both MSF and Ministry of Health health centre staff.

^2^ GeneXpert machine, cold chain and other medical equipment used in the field.

**^3^** Labour costs include both MSF and government salaries.

^4^ Renting of two cars and drivers for transport of active case finding team members and collected blood samples.

^5^ Rapid diagnostic tests, viral load tests and other materials for blood collection and information, education and communication materials.

^6^ MSF covered costs for uninsured patients to access tests other than HCV screening.

^7^ Overhead costs (salaries, administration costs and other office operation costs).

## Discussion

The two HCV screening strategies tested in Maung Russey operational district in Cambodia detected 1194 (5%) seropositive cases, 793 (66%) of whom were viraemic. The programme cost a total of US$ 184 941, with a cost per case screened of US$ 194 for passive screening and US$ 283 for combined passive and active screening. To our knowledge, this is the first HCV screening intervention based in primary health care centres and at the community level in Cambodia. The findings provide reliable evidence for policy and actions to scale up HCV case-finding in Cambodia.

Screening for HCV was voluntary, and 24 230 people were tested between March and December 2018 (18% of 133 162 targeted population). This relatively low screening rate might be due to low awareness of HCV in the community. A recent cross-sectional sero-survey reported that more than 64% of respondents were unaware of HCV. (*6*) Moreover, the asymptomatic nature of HCV infection might mean that people do not seek testing. Our screening programme did not include health promotion; however, information on HCV screening and treatment was provided to all eligible patients during their health centre visit. The low coverage of active screening might be due to the fact that screening was conducted on only one day per village, so that people who were not present on that day missed the opportunity. Extending the number of community screening days, a strategy that has been used in other countries such as Rwanda, (*12*) might increase participation. It is thus recommended that the national programme conduct active community screening for several days or organize annual catch-up screening campaigns.

We found that active screening greatly increased community screening coverage. While passive screening at health centres may reach symptomatic patients and those with known HCV status, active screening targets people outside health centres. Egypt, Rwanda and China, Taiwan (China) have implemented mass screening campaigns for the general population at community facilities such as stadiums and schools. (*11*, *12*, *14*) However, the entire general population was screened during such mass campaigns, while in this study active case finding was conducted only among people aged (*3*)45 years, who are known to be at high risk. (*6*) In Cambodia, use of other locations for reaching older people and other high-risk groups might be more affordable and feasible. (*15*, *16*) We also found that the NNS for finding one HCV case is lower when high-risk groups are targeted.

While the project was expected to achieve a higher seropositivity rate through active screening of adults aged (*3*)45 years, the rate in the same age group was higher with passive screening. The probable explanation is that people visiting health centres are either aware of their HCV status or are symptomatic or at a late stage and seeking care. Again, the higher positivity rate among older people supports active screening of these groups in a later phase of the project.

We considered seven categories of costs, some of which can be removed from future costing by changing the way screening is conducted. For example, integrating HCV screening with TB screening at all levels (health centres, district, provincial and national) would greatly reduce costs, such as for installing GeneXpert, transport and overhead. The cost of combined passive screening and active screening (US$ 283) was only US$ 89 higher than that for passive screening alone (US$ 194) but yielded a higher viraemic case detection rate (41% versus 28% viraemic cases, respectively). Moreover, the cost per viraemic case detected was comparable to the estimated cost per TB case, which was US$ 249 for active door-to-door screening in poor urban areas of Phnom Penh,  US$ 308 for testing of TB contacts and US$ 316 for symptomatic testing of the older rural population. (*17*) Integration of HCV screening with community programmes such as for TB, HIV and malaria could improve health workforce capacity and reduce the national cost of different screening programmes. (*14*) Another possible cost-saving strategy would be to take advantage of community gatherings during national holidays, such as the water festival and Pchum Ben, as well as World Hepatitis Day (28 July), to conduct periodic mass screening campaigns and HCV-related education.

### Strengths and limitations

A major strength of this study is that the output and cost data were collected and analysed with input from a field implementation programme. It thus captured real-world screening outcomes and costs. Moreover, having one implementing partner simplified the cost estimates.

A limitation of our study is that it is based on field experience and was not initially designed as operational research; it therefore relied on pooled data collected during the screening period. Moreover, we did not use common measures of effectiveness, such as quality-adjusted or disability-adjusted life-years. The comparability of these findings with those of other studies is thus limited.

## Conclusion

The combined passive and active screening approach cost US$ 89 more than passive screening alone yet provided a higher yield of viraemic cases (41% versus 28%, respectively). Thus, adding active screening to passive screening should be considered, as it might contribute to the objective of HCV elimination by 2030, as recommended by WHO.

### Contributors

JPD, MLP and IP conceived the study. SMH and IP managed the data and did the statistical analysis. IP checked for consistency of the analysis. SMH wrote the first draft of the article, which was edited for consistency by JPD, MLP, IP, CS and KA and critically revised for important intellectual content by CS and KA. All authors reviewed and approved the final version.

### Ethical considerations

The study received ethical approval from the Cambodian National Ethics Committee for Health Research, with reference no. 023 NECHR dated 7 February 2019.

As the data presented were collected as part of routine programmatic work and were anonymized, the protocol of the study was approved by MSF as fulfilling the exemption criteria set by the MSF Ethics Review Board for retrospective analyses of routinely collected data.
